# Arginine derivatives in atrial fibrillation progression phenotypes

**DOI:** 10.1007/s00109-020-01932-9

**Published:** 2020-06-06

**Authors:** Petra Büttner, Martin Bahls, Rainer H. Böger, Gerhard Hindricks, Holger Thiele, Edzard Schwedhelm, Jelena Kornej

**Affiliations:** 1 Department of Cardiology, Heart Center Leipzig at University Leipzig, Strümpellstrasse 39, 04289 Leipzig, Germany; 2 Department of Internal Medicine B, University Medicine Greifswald, 17475 Greifswald, Germany; 3 DZHK (German Centre for Cardiovascular Research), Partner Site Greifswald, 17475 Greifswald, Germany; 4 Institute of Clinical Pharmacology and Toxicology, University Medical Center Hamburg- Eppendorf, 20251 Hamburg, Germany; 5 DZHK (German Centre for Cardiovascular Research), Partner Site Hamburg/Kiel/Lübeck, 20251 Hamburg, Germany; 6 Department of Electrophysiology, Heart Center Leipzig at University Leipzig, 04289 Leipzig, Germany; 7 School of Medicine – Cardiovascular Medicine, Boston University, Boston, USA

**Keywords:** Atrial fibrillation, Progression phenotypes, Arginine, Homoarginine, ADMA, SDMA

## Abstract

**Abstract:**

Arginine, homoarginine (hArg), asymmetric dimethylarginine (ADMA), and symmetric dimethylarginine (SDMA) affect nitric oxide metabolism and altered concentrations are associated with cardiovascular morbidity and mortality. We analyzed these metabolites using liquid chromatography–tandem mass spectrometry in patients with atrial fibrillation (AF) (*n* = 241) with a focus on heart rhythm at blood withdrawal, AF progression phenotypes, and successful sinus rhythm (SR) restoration (*n* = 22). AF progression phenotypes were defined as paroxysmal AF with/without low voltage areas (LVA) and persistent AF with/without LVA. While arginine, ADMA, and hArg were within reference limits for healthy controls, SDMA was higher in the AF cohort (0.57 ± 0.12 vs. 0.53 μmol/L (97.5th percentile in reference cohort)). SR restoration in AF patients resulted in normalization of SDMA concentrations (0.465 ± 0.082 vs. 0.570 ± 0.134 μmol/L at baseline, *p* < 0.001). Patients with AF at the time of blood sampling had significantly lower hArg (1.65 ± 0.51 vs. 1.85 ± 0.60 μmol/L, *p* = 0.006) and higher ADMA concentrations (0.526 ± 0.08 vs. 0.477 ± 0.08 μmol/L, p < 0.001) compared with AF patients in SR. hArg concentrations were lower in patients with advanced AF progression phenotypes (persistent AF with LVA (*p* = 0.046)) independent of heart rhythm at blood sampling. Summarizing, arginine metabolism imbalance is associated with AF in general and AF progression and may contribute to associated risk.

**Key messages:**

• Heart rhythm at blood withdrawal affects ADMA and hArg level in AF patients.

• SDMA is higher in AF patients.

• SDMA levels normalize after sinus rhythm restoration.

• hArg levels decrease in advanced AF progression phenotypes.

**Electronic supplementary material:**

The online version of this article (10.1007/s00109-020-01932-9) contains supplementary material, which is available to authorized users.

## Introduction

Atrial fibrillation (AF) is the most common sustained arrhythmia worldwide [1]. The progression of AF can be characterized by a switch from paroxysmal (PAF) to persistent AF (persAF) [1], and peri-procedural evidence of low voltage areas (LVA) representing pro-fibrotic advanced left atrial (LA) remodeling processes [2, 3]. The progression of AF is associated with worse therapeutic outcomes [2–4] resulting in intensive health care costs because of higher hospitalization and greater complication rates.

LA remodeling is associated with impaired myocardial mechanics and unfavorable pathophysiological conditions such as oxidative stress [5]. Oxidative stress leads to reduced bioavailability of the important messenger molecule nitric oxide (NO), which plays a significant role in cardiovascular diseases [6], and especially in AF [7]. Impaired NO signaling is closely related to endothelial dysfunction, which is also observed in patients with AF [8]. Although NO is synthesized mostly from arginine, homoarginine (hArg) is an alternative source. Asymmetric dimethylarginine (ADMA) and symmetric dimethylarginine (SDMA) are irreversibly post-translationally modified arginine derivatives and inhibit NO synthase (NOS) through direct or indirect mechanism [9] (supplementary Fig. 1). Higher ADMA and SDMA as well as lower hArg concentrations were found to be associated with an increased cardiovascular mortality risk [10–12]. Furthermore, increased ADMA concentrations are associated with atherosclerosis, renal failure, hypertension, and hypercholesterolemia [13], which are considered common risk factors predisposing for cardiovascular diseases and AF [14]. Consequently, it was hypothesized that ADMA and SDMA concentrations might be associated with new onset of AF, but a sub-analysis in the community-based Framingham Heart Study could not confirm this relation [11]. Nevertheless, arginine metabolites could be of importance in AF initiation and progression as these are associated with endothelial dysfunction pointing towards a potential involvement of NO signaling [8]. Recently, we introduced a novel concept of AF progression phenotypes based on AF type and presence of LVA [15, 16], which can help to define pathomechanisms underpinned in the AF progression. We used this definition to show that patients with PAF without LVA have better biomarker and imaging profiles compared with patients with persAF and LVA [15, 16].

Restoration of SR in AF patients was also found to ameliorate endothelial function in AF patients [8, 17]. Therefore, in this study, we tested associations between NO synthesis-related arginine and its derivatives hArg, ADMA, and SDMA with (1) heart rhythm at blood withdrawal (SR or AF), (2) AF progression phenotypes, and (3) the impact of SR restoration.

## Materials and methods

### Patient population

The AF cohort was recruited from October 2015 until April 2017 including consecutively selected AF patients undergoing their first AF radiofrequency catheter ablation at the Heart Center Leipzig at the University of Leipzig (Leipzig, Germany) as previously described [15]. Exclusion criteria were pregnancy, age < 18 or > 75 years, valvular AF, current cancer, acute, or systemic inflammatory diseases. PAF and persAF were defined according to current guidelines [18]. PAF was defined as self-terminating within 48 h–7 days after onset. PersAF lasted longer than 7 days or required drugs or direct current cardioversion for termination. Patients with long-standing persistent or permanent AF were not included as AF management in these patients was based on rate control and oral anticoagulation. Serum creatinine concentrations were assessed before ablation. Estimated glomerular filtration rate (eGFR) was calculated using the CKD-EPI (Chronic Kidney Disease Epidemiology Collaboration) equation: eGFR = 141 × min(Scr/*ĸ*, 1)*α* × max(Scr/*ĸ*, 1) − 1.209 × 0.993 age × 1.018 [if female] × 1.159 [if black], where Scr is serum creatinine, *ĸ* is 0.7 for females and 0.9 for males, *α* is − 0.329 for females and − 0.411 for males, min indicates the minimum of Scr/ĸ or 1, and max indicates the maximum of Scr/*ĸ* or 1 [19]. Coronary artery disease, peripheral vascular disease, and atherosclerotic plaques were summarized as a vascular disease.

The cohort was initially recruited to detect an association of NT-proANP with LVA in AF patients [15]. Based on previous power calculations, 250 participants were suitable to detect at least an effect size of 0.4 (given *α* = 0.05, *β* = 0.8). Out of these 250 participants, nine had to be excluded due to insufficient sample quality or volume. We used all available samples (*n* = 241) for the secondary analyses reported herein.

The study was approved by the local Ethical Committee (Medical Faculty, University Leipzig, IRB No.: 259-15-13072015) and all patients provided written informed consent for participation in accordance with the Declaration of Helsinki.

### AF progression phenotypes

We used standardized clinical AF phenotypes to build AF cohort subgroups: (1) LVA detection was performed peri-interventionally during AF catheter ablation and we compared patients with and without LVA [2, 3]; (2) we used LVA in combination with AF type (PAF vs. persAF) to stratify four phenotypes (PAF without LVA, persAF without LVA, PAF with LVA, and persAF with LVA). These four groups were regarded as stepwise increasing progression phenotypes whereas patients with PAF without LVA are considered more healthy and have favorable outcomes after catheter ablation, while patients with persAF and LVA have more advanced phenotype and unfavorable outcomes [15].

### Catheter ablation procedure

Catheter ablation procedure with isolation of the pulmonary veins and additional lesions if required was performed as reported previously [15] (see supplementary materials and methods). Briefly, the electro-anatomical mapping was performed in SR. In patients who presented with AF prior ablation procedure, the arrhythmia was terminated by electrical cardioversion and the procedure was further performed in SR. End point of the catheter ablation was isolation of the pulmonary veins with proof of both exit and entrance block. In both mapping systems, the cutoff values for defining LVA were identical: < 0.5 mV for low voltage and < 0.2 mV for dense scar.

### Blood samples

Blood samples from AF patients were obtained in EDTA tubes after fasting > 8 h from the femoral vein before trans-septal puncture and left atrial ablation. At follow-up examinations, blood was withdrawn from the cubital vein. Samples were processed within 1 h of collection. Blood plasma was prepared (1000×*g* for 10 min at 20 °C) and aliquots were stored at − 70 °C for subsequent analysis.

### Determination of arginine, hArg, ADMA, and SDMA

Established and validated protocols for liquid chromatography–tandem mass spectroscopy (LC-MS/MS) were used to assess plasma arginine, ADMA, SDMA, and hArg concentrations [20–22] (see supplementary materials and methods). Briefly, 25 μL of plasma was diluted in methanol with stable isotope labeled internal standards. Thereafter, the analytes were converted into their butyl esters. Analyte concentrations were calculated using calibration curves based on four levels in triplicates. Plate-wise quality controls (QC) were run in two levels by triplicates. A second analysis was done on the samples to assess coefficient of variation and bias of QC, which had to be below 15%.

### Data analysis and statistics

Data were analyzed for outliers by transformation into *Z*-scores and exclusion of values > 3 standard deviations. Respectively, two values for arginine and three values for ADMA, SDMA, and hArg were removed from further analysis. Continuous variables were tested for normal distribution using the Kolmogorov-Smirnov test and found to be not normally distributed. The differences between continuous values were assessed using Mann-Whitney *U* test (two groups), Kruskal Wallis test (more than two groups), and Wilcoxon exact test was used for before-after ablation comparisons. Chi-square test was used for categorical variables. Correlations were analyzed using Spearman’s rank-order correlation.

We analyzed arginine derivative concentrations in AF progression using different approaches. First, we compared the concentrations in patients with and without LVA and secondly, we used the concept of four AF progression stages (Table 1). In a first step, univariate regression models were used to identify relationships between arginine, ADMA, SDMA, and hArg with age, sex, type of AF, LVA, BMI, eGFR, hypertension, diabetes type 2, LA diameter, and heart rhythm at baseline (Table 2). Thereafter, the significantly related parameters were included in multivariate regression model. A *p* value < 0.05 was considered statistically significant. All analyses were performed with IBM SPSS Statistics for Windows version 25 (IBM Corp, Armonk, NY, USA), SPSS statistical software version 23, and GraphPad Prism 8 (GraphPad Software, San Diego, CA, USA).

**Table 1:** Clinical characteristics of study cohort (*n* = 241) and concentration of hArg, ADMA, and SDMA

	Total	SR	AF	*p* value	No LVA	LVA	*p* value	PAF w/o LVA	persAF w/o LVA	PAF LVA	persAF LVA	p value
*n* (%)	241	111 (46)	128 (54)		176 (73)	65 (27)		86 (36)	90 (37)	12 (5)	53 (22)	
Age (years)	64 ± 11	62 ± 12	65 ± 9	0.041	62 ± 11	69 ± 7	< 0.001	63 ± 12	61 ± 11	69 ± 7	69 ± 7	< 0.001
Women (*n* (%))	98 (41)	45 (41)	52 (41)	1.000	62 (35)	36 (55)	0.005	38 (44)	24 (27)	7 (58)	29 (55)	0.104
Persistent AF (*n* (%))	143 (59)	37 (33)	103 (81)	< 0.001	90 (51)	53 (82)	< 0.001	-	-	-	-	-
LVA (*n* (%))	65 (27)	19 (29)	45 (69)	0.006	-	-	-	-	-	-	-	-
BMI (kg/m^2^)	30 ± 5	29 ± 6	30 ± 5	0.659	29 ± 6	30 ± 5	0.415	29 ± 6	30 ± 5	30 ± 4	30 ± 5	0.200
eGFR (mL/min/1.73 m^2^)	76 ± 18	77 ± 20	75 ± 17	0.369	79 ± 18	68 ± 17	< 0.001	80 ± 18	78 ± 18	69 ± 23	68 ± 16	0.001
Hypertension, n (%)	196 (81)	86 (78)	109 (85)	0.135	133 (76)	63 (97)	< 0.001	60 (70)	73 (81)	12 (100)	51 (96)	< 0.001
Diabetes (*n* (%))	54 (22)	16 (14)	38 (30)	0.005	31 (18)	23 (35)	0.003	11 (13)	20 (22)	0	23 (43)	< 0.001
Vasc. disease (*n* (%))	52 (22)	23 (21)	29 (23)	0.709	30 (17)	22 (34)	0.008	16 (19)	14 (16)	6 (50)	16 (30)	0.015
LA diameter (mm)	44 ± 7	26 ± 6	30 ± 7	< 0.001	43 ± 7	45 ± 6	0.044	41 ± 6	45 ± 6	43 ± 7	46 ± 6	< 0.001
SR at baseline (*n* (%))	111 (46)	-	-	-	92 (52)	19 (29)	0.006	66 (77)	26 (29)	7 (58)	12 (23)	0.003
CHA_2_DS_2_-VASc score	3 (1–4)	2 (1–4)	3 (2–4)	0.111	2 (1–4)	3 (3–4)	< 0.001	2 (1–4)	2 (1–3)	3 (3–4)	4 (3–5)	< 0.001
Arginine (μmol/L)	66 ± 16	67 ± 18	65 ± 14	0.310	67 ± 16	63 ± 15	0.120	68 ± 17	66 ± 15	59 ± 14	64 ± 15	0.246
hArg (μmol/L)	1.74 ± 0.6	1.85 ± 0.6	1.65 ± 0.5	0.006	1.80 ± 0.6	1.58 ± 0.4	0.001	1.84 ± 0.7	1.76 ± 0.5	1.5 ± 0.4	1.59 ± 0.4	0.038
ADMA (μmol/L)	0.50 ± 0.1	0.48 ± 0.1	0.53 ± 0.1	< 0.001	0.50 ± 0.1	0.51 ± 0.8	0.219	0.48 ± 0.1	0.51 ± 0.1	0.46 ± 0.1	0.53 ± 0.1	0.004
SDMA (μmol/L)	0.57 ± 0.1	0.56 ± 0.1	0.58 ± 0.1	0.135	0.56 ± 0.1	0.58 ± 0.1	0.104	0.56 ± 0.1	0.56 ± 0.1	0.54 ± 0.1	0.59 ± 0.1	0.064

*SR* sinus rhythm, *AF* atrial fibrillation, *LVA* low voltage areas, *PAF* paroxysmal atrial fibrillation, *persAF* persistent atrial fibrillation, *w/o* without, *eGFR* estimated glomerular filtration rate, *BMI* body mass index, *LA* left atrial, *hArg* homoarginine, *ADMA* asymmetric dimethylarginine, *SDMA* symmetric dimethylarginine, *p* values ​​relate to the comparison of the two previous columns, the *p* value in the last column relates to the comparison of the four *AF* progression stages

Data are provided as mean ± standard deviation or median (interquartile range) and *n* (%)

**Table 2:** Multivariate (MV) regression analyses for arginine, hArg, ADMA, and SDMA. Beta coefficients and *p* values are presented. UV—results from univariate regression analysis. Parameters entered the MV when UV *p* value < 0.05

	Arginine		hArg				ADMA				SDMA			
	UV		UV		MV		UV		MV		UV		MV	
	Beta	*p* value	Beta	*p* value	Beta	*p* value	Beta	*p* value	Beta	*p* value	Beta	*p* value	Beta	*p* value
Age (years)	− 0.051	0.432	− 0.179	0.006	0.119	0.135	0.223	0.001	0.193	0.012	0.399	< 0.001	0.069	0.313
Women	− 0.099	0.127	− 0.293	< 0.001	− 0.309	< 0.001	0.071	0.277			0.122	0.087		
Persistent AF	0.039	0.552	0.098	0.132			− 0.208	0.001	− 0.074	0.336	− 0.058	0.376		
LVA	− 0.103	0.111	− 1.76	0.007	− 0.093	0.147	0.078	0.231			0.071	0.280		
BMI (kg/m^2^)	0.052	0.421	0.216	0.001	0.292	< 0.001	0.131	0.045	0.126	0.086	− 0.029	0.654		
eGFR (mL/min)	0.087	0.180	0.216	0.001	0.141	0.065	− 0.097	0.136			− 0.586	< 0.001	− 0.539	< 0.001
Hypertension	0.046	0.481	0.053	0.420			0.191	0.003	0.074	0.324	0.171	0.009	0.026	0.641
Diabetes	− 0.009	0.894	0.051	0.437			0.188	0.004	0.059	0.390	0.108	0.098		
Vascular disease	− 0.056	0.392	− 0.025	0.700			− 0.069	0.289			0.103	0.113		
LA diameter (mm)	0.011	0.877	− 0.006	0.927			0.184	0.007	0.109	0.123	− 0.066	0.334		
SR at baseline	0.025	0.730	0.176	0.007	0.143	0.026	− 0.290	< 0.001	− 0.218	0.002	− 0.074	0.255		

Abbreviations: as in Table 1

## Results

The baseline study cohort characteristics are presented in Table 1. In total, 241 patients (64 ± 11 years, 59% males, BMI 30 ± 5 kg/m^2^, 41% PAF, 27% with LVA) were included. Patients with LVA were older, more often women, had more often persAF, hypertension, diabetes, vascular disease, lower eGFR, LA diameter, and CHAD_2_DS_2_-VASc score (Table 1). Patients with more advanced AF progression phenotypes (persAF with LVA) were older, had more comorbidities (hypertension, diabetes, vascular disease) and higher eGFR, LA diameter, and CHAD_2_DS_2_-VASc score than patients with PAF without LVA. While there were no significant sex-specific differences in ADMA and SDMA, hArg concentration was significantly higher in men than in women (1.88 ± 0.59 μmol/L vs.1.54 ± 0.46 μmol/L, *p* < 0.001). Similarly, arginine concentration was higher in men compared with women (66.9 ± 14.9 μmol/L, 63.7 ± 16.9 μmol/L, *p* = 0.019).

### Arginine, ADMA, SDMA, and hArg concentrations and heart rhythm at blood withdrawal

The impact of heart rhythm at blood sampling was examined in AF patients presenting with AF (*n* = 128) or SR (*n* = 111) at the time of blood withdrawal. Patients in SR had significantly higher hArg (1.85 ± 0.60 vs. 1.65 ± 0.51, *p* = 0.006) and lower ADMA (0.477 ± 0.08 vs. 0.526 ± 0.08, *p* < 0.001). Heart rhythm was not related to arginine and SDMA concentration (Table 1).

### Association of clinical parameters with arginine, ADMA, SDMA, and hArg

Results of the univariate and multivariate regression analyses are presented in Table 2. We found that female sex, lower BMI, and AF at blood withdrawal were associated with lower hArg. BMI was correlated with hArg (Spearman’s *r* = 0.195, *p* = 0.003). Older age and AF at blood withdrawal were independent predictors of higher ADMA concentrations in multivariate analysis. Age correlated significantly with ADMA (Spearman’s *r* = 0.166, *p* = 0.01).

For SDMA, only eGFR was significantly associated in multivariate analysis (Table 2). SDMA and eGFR were inversely correlated (Spearman’s *r* = − 0.602, *p* < 0.0001). No associations were found for arginine in multivariate regression analysis.

### Arginine, ADMA, SDMA, and hArg in AF progression phenotypes

Arginine was not associated with LVA or progression phenotypes. hArg concentrations were lower in patients with LVA (1.58 ± 0.42 vs. 1.80 ± 0.60 μmol/L, *p* < 0.001 (Supplemental Fig. 2)) and in advanced AF progression stages (*p* = 0.038). ADMA and SDMA concentrations were not associated with LVA but were higher in advanced AF progression stages (*p* = 0.004 and *p* = 0.064, respectively) (Supplemental Fig. 2).

Due to a strong association between heart rhythm at blood withdrawal with hArg and ADMA concentrations as well as significant differences in sub-cohort characteristics, we used multivariate analysis to prove the association of arginine derivatives with LVA and AF progression stage (Table 3). In multivariate analysis, hArg was associated with AF progression stage (*p* = 0.046). There was no relationship between arginine, ADMA, and SDMA concentrations and LVA or AF progression phenotypes.

**Table 3:** Multivariate regression analyses for LVA and AF progression phenotypes. Odds ratios (95% CI) for LVA, beta coefficients for AF progression phenotypes, and *p* values are presented. UV—results from univariate analysis. Parameters entered the MV when UV *p* value < 0.05

	LVA*				AF progression phenotypes^#^			
	UV		MV		UV		MV	
	OR (95% CI)	*p* value	OR (95% CI)	*p* value	OR (95% CI)	*p* value	OR (95% CI)	*p* value
Age (years)	1.08 (1.04–1.12)	< 0.0001	1.09 (1.03–1.15)	0.002	1.04 (1.01–1.06)	0.002	1.01 (0.97–1.04)	0.682
Women	2.45 (1.35–4.45)	0.003	2.88 (1.29–6.47)	0.01	1.29 (0.80–2.10)	0.293		
Persistent AF	0.435 (0.234–0.808)	0.008	0.604 (0.27–1.33)	0.212	-	-		
BMI (kg/m^2^)	1.03 (0.979–1.08)	0.250			1.03 (0.99–1.08)	0.144		
eGFR (mL/min)	0.972 (0.955–0.989)	0.001	1.01 (0.98–1.03)	0.705	0.98 (0.96–0.99)	< 0.001	0.98 (0.96–1.00)	0.078
Hypertension	9.32 (2.18–39.77)	0.003	3.62 (0.77–17.10)	0.105	3.84 (2.01–7.39)	< 0.001	2.74 (1.23–6.10)	0.014
Diabetes	2.27 (1.17–4.39)	0.015	1.28 (0.58–2.84)	0.547	2.71 (1.54–4.81)	0.001	1.38 (0.71–2.70)	0.341
Vascular disease	2.49 (1.30–4.75)	0.06			1.72 (0.96–3.06)	0.066		
LA diameter (mm)	1.06 (1.01–1.11)	0.011	1.09 (1.02–1.16)	0.012	1.11 (1.07–1.16)	< 0.001	1.09 (1.05–1.14)	< 0.001
SR at baseline	0.373 (0.200–0.696)	0.02	0.622 (0.28–1.40)	0.239	0.21 (0.13–0.35)	< 0.001	0.26 (0.14–0.49)	< 0.001
Arginine (μmol/L)	0.985 (0.966–1.004)	0.122			0.99 (0.98–1.01)	0.224		
hArg (μmol/L)	0.448 (0.247–0.809)	0.08			0.57 (0.37–0.89)	0.013	0.58 (0.34–0.99)	0.046
ADMA (μmol/L)	7.86 (0.27–229.33)	0.231			47.47 (2.78–810.78)	0.008	1.32 (0.03–55.37)	0.883
SDMA (μmol/L)	3.58 (0.354–36.29)	0.280			9.27 (1.58–54.43)	0.014	0.58 (0.04–9.09)	0.695

Abbreviations: as in Table 1

*Logistic regression analysis with LVA as dependent variable

^#^Ordinal regression analysis with AF progression phenotypes (paroxysmal AF with/without low voltage areas (LVA) and persistent AF with/without LVA) as dependent variable

### Arginine, ADMA, SDMA, and hArg in AF patients with restored SR

**Fig. 1:**
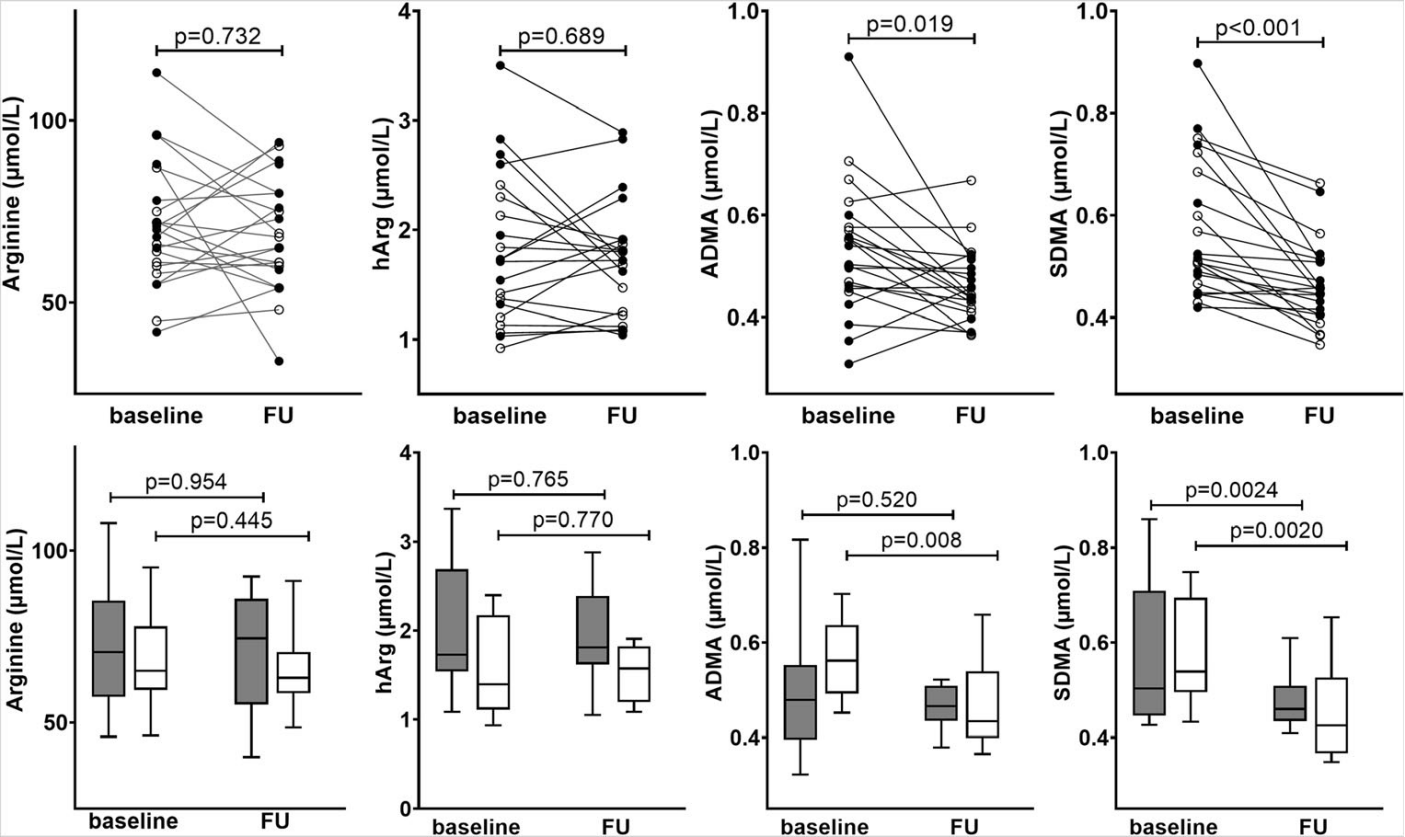
Paired measurements of Arginine, hArg, ADMA, and SDMA in AF patients before (baseline) and after catheter ablation procedure (FU) with successful sinus rhythm (SR) restoration. Upper panels show individual values while black circles indicate patients with SR at baseline blood withdrawal and white circles indicate patients with AF at baseline blood withdrawal. For the upper panels, *p* values were calculated for all patients irrespective of heart rhythm at baseline blood withdrawal. Lower panels show group-wise medians with whiskers 10–90 percentiles and group-wise *p* values for patients in SR at baseline blood withdrawal (dark gray boxes, upper *p* value) or in AF (white boxes, lower *p* value)

There were 22 AF patients with restored SR after catheter ablation and available blood samples at 12–18 months follow-up (FU). SDMA concentrations of these 22 AF patients with restored SR at FU were 0.465 ± 0.082 μmol/L compared with 0.570 ± 0.134 μmol/L at baseline (*p* < 0.01, Fig. 1). This observation was independent of the heart rhythm at the time point of baseline blood sampling. The eGFR was similar at baseline and FU (84 ± 14 vs. 83 ± 15, respectively, *p* = 0.482). In patients with SR at baseline and follow-up arginine, ADMA and hArg concentrations did not change over time. ADMA concentrations were significantly lower at FU when patients were in AF at blood withdrawal (*p* = 0.008).

## Discussion

AF patients represent a relatively heterogeneous group with complex comorbidities, e.g., hypertension, diabetes, and coronary artery disease resulting in individual AF patterns. In addition, the underpinning pathomechanisms are heterogeneous including pro-fibrotic and pro-inflammatory processes that lead to electro-anatomical remodeling of the atrial myocardium whereas advanced changes are associated with poorer therapeutic success [2, 3, 23]. This is challenging for the development of standardized therapies or identification of underlying pathomechanisms [24]. Recently, we introduced a novel concept to differentiate AF patients using four AF progression phenotypes and demonstrated significant differences in blood biomarkers and imaging profiles dependent on AF progression stages [15, 16]. In our current study, we analyzed associations between AF progression phenotypes with arginine and its derivatives ADMA and SDMA as well as the non-proteinogenic amino acid hArg in patients with AF undergoing catheter ablation. Both arginine and ADMA concentrations were within the reference values of 41 and 114 μmol/L (2.5th and 97.5th percentile) and 0.311 and 0.732 μmol/L, respectively, based on our data from the Framingham Offspring Study [25]. The mean SDMA plasma concentrations in our clinical AF cohort were 0.57 ± 0.12 μmol/L and thus above the SDMA reference values 0.225 and 0.533 μmol/L (2.5th and 97.5th percentile) determined previously [26]. Finally, the mean hArg concentrations were within the sex-specific references in males (1.88 ± 0.59 μmol/L (2.5th and 97.5th percentile 0.98 and 4.10 μmol/L)) and females (1.54 ± 0.46 μmol/L (2.5th and 97.5th percentile 0.84 and 3.89 μmol/L)) as determined using the same methodological platform in healthy participants (no CV disease or risk factors) of the Gutenberg Health Study [27]. We found hArg concentrations to be associated with sex and BMI, which is in accordance with previous research [28]. ADMA concentrations were associated with higher age as reported before [21]. The strong association of low eGFR with high SDMA was also previously known [29].

An important finding of our study was that heart rhythm during blood withdrawal per se was associated with arginine derivatives. hArg concentrations were 11% lower and ADMA concentrations were 10% higher in patients with AF when compared with patients in SR at the time of blood sampling. These findings are in accordance with previous research [30], where rapid atrial pacing for 7 h in an animal model increased ADMA concentrations, while simultaneously mRNA levels of ventricular and aortic endothelial NOS decreased [30]. In humans, SR restoration after cardioversion resulted in normalization of ADMA concentrations within 24 h [30]. Considering our study design, we were not able to detect short-termed ADMA concentration decreases in our cohort as follow-up blood was available only 12–18 months after ablation. While ADMA inhibits NOS, the parallel depletion of hArg that we observed may additionally contribute to a suspected NO dysbalance, as hArg is an alternative substrate for NO synthesis [11]. Noteworthy, many studies that focus on arginine derivatives in AF do not report heart rhythm at blood withdrawal and consequently do not consider it as a potentially important variable influencing biomarker levels. Our results indicate the importance to analyze heart rhythm as this variable seems to influence the biomarker’s impact in AF pathogenesis. Further studies should describe in detail the acute concentration changes of arginine derivatives in response to AF episodes.

hArg was independently associated with AF progression phenotypes after adjustment for heart rhythm and sub-cohort differences in multivariate analysis. Recent results from genome wide association studies identified a strong link between plasma hArg concentrations and the enzyme L-arginine:glycine amidinotransferase (AGAT, *GATM* gene locus) [31, 32], which is mainly expressed and functionally relevant for the kidney [33], brain [34], and to some extent the heart [35]. Likewise, mice with genetic deletion of AGAT exhibit an exacerbated experimental stroke and heart failure phenotype [36, 37]. In addition to the anabolism of hArg, AGAT is also the rate-limiting enzyme in creatine biosynthesis [37]. Creatine represents an energy buffer in skeletal muscle, brain, heart, and several other high energy demand tissues. Especially in AF, cardiomyocytes are exposed to high-frequency excitation and contraction resulting in increased energy and oxygen demand and increased metabolic stress [38]. The ATP generation by actomyosin-ATPase in the cardiac myofilaments thus increases while the enzyme is highly depending on creatine [38]. AGAT thus may represent a potential target in AF pathomechanisms.

In our study, SDMA was generally higher in patients with AF, but was not associated with AF progression. In contrast to hArg and ADMA, SDMA concentrations were not affected by acute changes in heart rhythm. We thus assumed that SDMA increase is rather a long-term consequence of AF. In line with this assumption, we found that SDMA significantly decreased following SR restoration below the reference limit of 0.533 μmol/L (97.5th percentile) determined for healthy individuals. We observed a correlation of SDMA with eGFR that is in line with recent findings [39]. Renal dysfunction is a common finding in AF patients [18]. In our analysis, there were only few patients with chronic kidney disease (eGFR < 60 mL/min) and none with severe renal failure (stages IV and V). With regard to the cardiovascular risk that is associated with high SDMA concentrations [40], our findings highlight the importance for the prevention and timely diagnosis of renal dysfunction in AF patients. Interestingly, while eGFR did not improved during follow-up, SDMA still decreased. Thus, the SDMA increase in AF seems to be triggered by other effectors than renal dysfunction alone. Of note, SDMA was found to modify HDL particles thereby reducing HDL function [41, 42]. Recently, we demonstrated that HDL characteristics (cholesterol efflux capacity, HDL particle number, apoA-I levels, and lecithin–cholesterol acyltransferase activity) are markedly reduced in AF. Furthermore, SR restoration ameliorated HDL function [43]. These findings indicate a potential link between SDMA and HDL function in AF. Whether SDMA is a suitable marker to predict AF recurrences should be addressed in further studies considering potential covariates such as operators’ experience, consistent definition of AF recurrences, and inclusion of all patients in FU.

### Limitations

The use of AF progression phenotypes is an evidence-based model that helps to characterize AF sub-populations. We are well aware that individual AF progression not necessarily proceeds through the four progression stages and that particularly the patients with PAF and LVA may be an entity with specific risk factors and a different pathomechanistic background. Nevertheless, we found the concept very helpful for the characterization of AF progression–associated markers. The impact of smoking, the duration of AF, often complex individual medication, and other biomarkers were not analyzed in this study although they might be of importance. Also, our observations of arginine derivative concentrations in AF patients with restored SR are based only on a small subgroup, that is why interpretation of these results should be cautious. The potential of SDMA as a biomarker of AF recurrence should be examined in a larger cohort considering its connection to patient’s renal function, i.e., eGFR [44]. Nevertheless, our results are hypothesis-generating and contribute to the understanding of AF-associated risk and arginine derivatives as potential mediators.

## Conclusion

Heart rhythm at blood withdrawal affects ADMA and hArg concentrations in AF patients. Advanced AF progression phenotypes are associated with lower hArg concentration. SDMA is higher in AF and is ameliorated after SR restoration. Further studies are needed to evaluate the impact of hArg supplementation and/or lowering of ADMA/SDMA on AF progression.

## Electronic supplementary material

ESM 1 (DOCX 445 kb)

## Data Availability

Data is available from the authors on request.
