# Correction to: Arginine derivatives in atrial fibrillation progression phenotypes

**DOI:** 10.1007/s00109-021-02147-2

**Published:** 2021-10-30

**Authors:** Petra Büttner, Martin Bahls, Rainer H. Böger, Gerhard Hindricks, Holger Thiele, Edzard Schwedhelm, Jelena Kornej

**Affiliations:** 1 Department of Cardiology, Heart Center Leipzig at University Leipzig, Strümpellstrasse 39, 04289 Leipzig, Germany; 2 Department of Internal Medicine B, University Medicine Greifswald, 17475 Greifswald, Germany; 3 DZHK (German Centre for Cardiovascular Research), Partner Site Greifswald, 17475 Greifswald, Germany; 4 Institute of Clinical Pharmacology and Toxicology, University Medical Center Hamburg- Eppendorf, 20251 Hamburg, Germany; 5 DZHK (German Centre for Cardiovascular Research), Partner Site Hamburg/Kiel/Lübeck, 20251 Hamburg, Germany; 6 Department of Electrophysiology, Heart Center Leipzig at University Leipzig, 04289 Leipzig, Germany; 7 School of Medicine – Cardiovascular Medicine, Boston University, Boston, USA

## Correction to: Journal of Molecular Medicine (2020) 98:999–1008 https://doi.org/10.1007/s00109-020–01,932-9

The article Arginine derivatives in atrial fibrillation progression phenotypes by Petra Büttner, Martin Bahls, Rainer H. Böger, Gerhard Hindricks, Holger Thiele, Edzard Schwedhelm and Jelena Kornej, was originally published online on 06 June 2020 without open access. With the author(s)’ decision to opt for Open Choice the copyright of the article changed on 27 September 2021 to © The Author(s) 2021 and the article is forthwith distributed under a Creative Commons Attribution 4.0 International License, which permits use, sharing, adaptation, distribution and reproduction in any medium or format, as long as you give appropriate credit to the original author(s) and the source, provide a link to the Creative Commons licence, and indicate if changes were made. The images or other third party material in this article are included in the article’s Creative Commons licence, unless indicated otherwise in a credit line to the material. If material is not included in the article’s Creative Commons licence and your intended use is not permitted by statutory regulation or exceeds the permitted use, you will need to obtain permission directly from the copyright holder. To view a copy of this licence, visit http://creativecommons.org/licenses/by/4.0.

The Original article has been corrected.

