# Supplementary material for: Arginine derivatives in atrial fibrillation progression phenotypes
Source: J Mol Med (Berl). 2020 Jun 6;98(7):999–1008. doi: 10.1007/s00109-020-01932-9 (PMC8556202; doi:10.1007/s00109-020-01932-9)
Supplement: Supplementary file 1 — (DOCX 445 kb) [file 109_2020_1932_MOESM1_ESM.docx]

**Supplementary data: Arginine Derivatives in Atrial Fibrillation Progression Phenotypes**

Petra Büttner^1^, PhD, Martin Bahls^2,3^, PhD, Rainer H. Böger^4,5^, MD, Gerhard Hindricks^6^, MD, Holger Thiele^1^, MD, *Edzard Schwedhelm^4,5^, PhD, *Jelena Kornej^7^, MD, MSc

**Supplementary Material and Methods**

*Catheter ablation procedure*

The electro-anatomical mapping was performed as previously described [1]. Briefly, two 3-D mapping systems (Carto, Biosense Webster, Diamond Bar, CA, USA or EnSite Precision, St Jude Medical (SJM), Saint Paul, MN, USA) were used for electro-anatomical mapping. The spiral mapping catheter used for NavX Ensite procedures was the Reflexion Spiral (SJM, Saint Paul, MN, USA) and the Carto Lasso (Biosense Webster, Diamond Bar, CA, USA) for Carto3 procedures. In both mapping systems, the cut-off values for defining LVAs were identical: <0.5mV for low voltage and >0.5mV for normal voltage. Patients underwent high-density mapping of left atrial voltage using multipolar catheters in combination with auto-annotation algorithms (AutoMap in Precision and ConfiDense in Carto 3). The voltage mapping points were obtained in sinus rhythm before ablation or after ablation of the pulmonary veins. Here the number of points was >1000. According to the presence of LVAs individually tailored ablation lines were added after the electro-anatomical map**.** Only patients with electro-anatomical substrate leading to additional linear ablations were considered as the LVAs group.

*Determination of arginine, hArg, ADMA and SDMA*

A stable isotope dilution assay for the liquid chromatography-tandem mass spectroscopy (LC-MS/MS) determination of arginine, hArg, ADMA and SDMA was applied which was developed, validated and published previously by the author’s lab [2,3]. For protein precipitation, a 96-well 0.2 µm microfiltration plate was place on top of a 96-well u-shaped polypropylene autosampler plate. Each well of the upper plate was filled with 2H7-arginine, 13C6-homoarginine, and 2H6-ADMA solved in 100 µL methanol and 25 µL EDTA plasma sample, calibrator, or quality control sample were added. Stable isotope labeled internal standard concentrations corresponding to concentrations of 40, 10 and 2 µM for the analytes arginine, hArg, ADMA and SDMA, respectively. 2H6-ADMA was used as internal standard for ADMA and SDMA. After centrifugation for protein precipitation by filtration and subsequent evaporation of the eluent to dryness by heating to 65 °C, the analytes were converted into their butyl esters derivatives with butanolic HCL at 85 °C. Analyte concentrations were calculated using calibration curves based on four levels in triplicates. The analytes were added to dialyzed plasma previously obtained by dialyzing 20 mL pooled human EDTA plasma with a Slid-A-Lyzer™ cassette against 1,5 L saline three times for 24 hours. The following calibration levels were used for the analytes. Argnine 0, 60, 120, and 250 µM. hArg 0, 2, 5, and 10 µM. ADMA 0, 0.5, 1, and 2 µM. SDMA 0, 0.5, 1, and 2 µM. Calibration curves for each analyte were generated by plotting the calibration level (x-axis) against the peak area ratios of the analyte divided by the internal standard (y-axis). The analyte concentration in a plasma EDTA or quality control sample was calculated by the corresponding peak area ratios divided by the slope of the calibration curve.

Methods references

1. Büttner, P.; Schumacher, K.; Dinov, B.; Zeynalova, S.; Sommer, P.; Bollmann, A.; Husser, D.; Hindricks, G.; Kornej, J. Role of NT-proANP and NT-proBNP in patients with atrial fibrillation: Association with atrial fibrillation progression phenotypes. Heart Rhythm 2018, 1132–1137, doi:10.1016/j.hrthm.2018.03.021.

2. Atzler, D.; Mieth, M.; Maas, R.; Böger, R.H.; Schwedhelm, E. Stable isotope dilution assay for liquid chromatography-tandem mass spectrometric determination of L-homoarginine in human plasma. J. Chromatogr. B Analyt. Technol. Biomed. Life Sci. 2011, 879, 2294–2298, doi:10.1016/j.jchromb.2011.06.016.

3. Schwedhelm, E.; Maas, R.; Tan-Andresen, J.; Schulze, F.; Riederer, U.; Böger, R.H. High-throughput liquid chromatographic-tandem mass spectrometric determination of arginine and dimethylated arginine derivatives in human and mouse plasma. J. Chromatogr. B Analyt. Technol. Biomed. Life Sci. 2007, 851, 211–219, doi:10.1016/j.jchromb.2006.11.052.


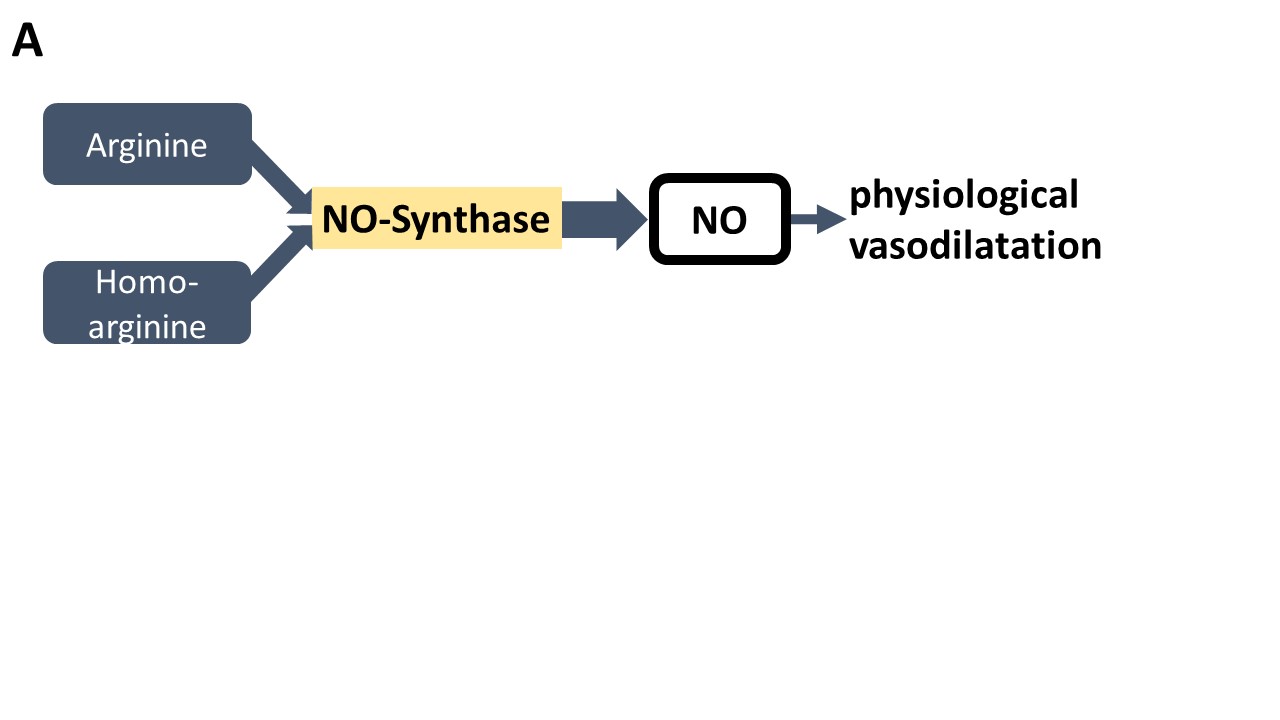


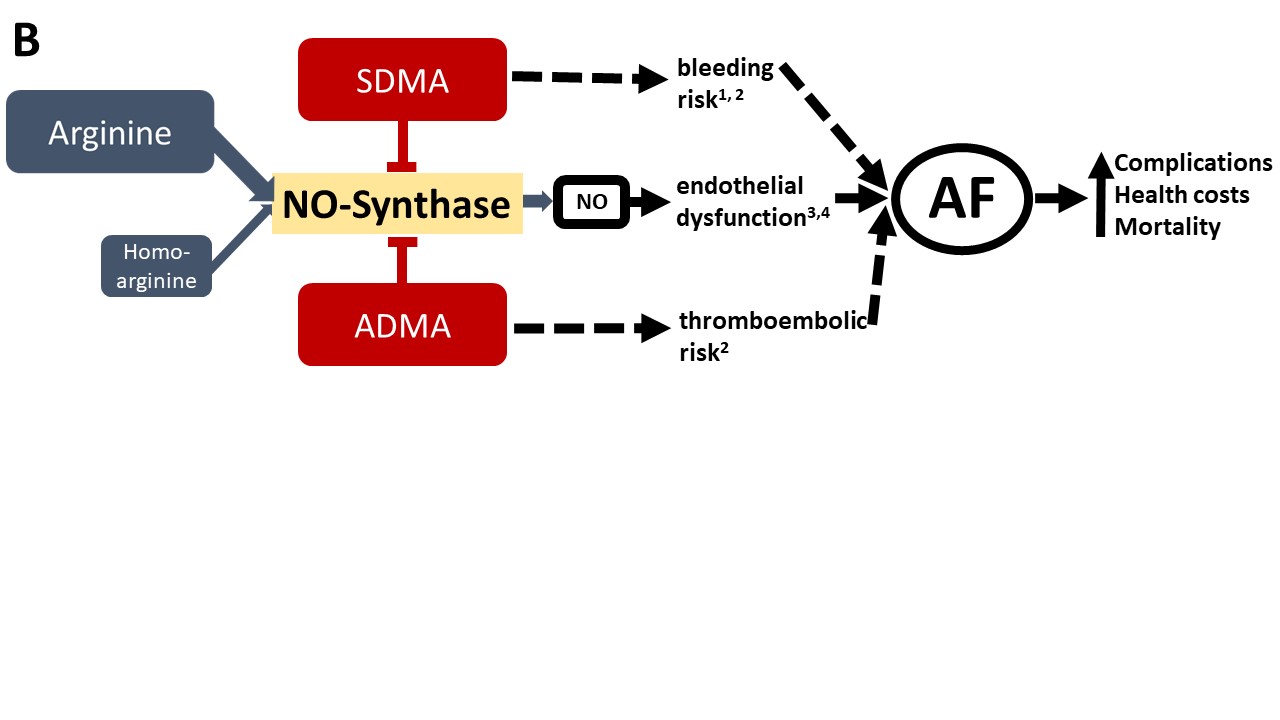


**Supplemental Figure 1:** Simplified scheme of the interactions of arginine, homo-arginine, ADMA and SDMA with the NO-Synthase: A - under physiological conditions. B – arginine derivatives and proposed related mechanism in atrial fibrillation (AF) patients. Blue boxes – substrates for NO-Synthase, red boxes – inhibitors of NO-Synthase. Dashed lines indicate pathomechanisms that were found to be associated with arginine derivatives and atrial fibrillation. ADMA – asymmetric dimethylarginine, SDMA – symmetric dimethylarginine.

Figure references:

1. Horowitz, J.D.; Caterina, R. de; Heresztyn, T.; Alexander, J.H.; Andersson, U.; Lopes, R.D.; Steg, P.G.; Hylek, E.M.; Mohan, P.; Hanna, M.; et al. Asymmetric and Symmetric Dimethylarginine Predict Outcomes in Patients With Atrial Fibrillation: An ARISTOTLE Substudy. J. Am. Coll. Cardiol. 2018, 72, 721–733, doi:10.1016/j.jacc.2018.05.058.

2. Ziegler, N.L.; Sieweke, J.-T.; Biber, S.; Gabriel, M.M.; Schuppner, R.; Worthmann, H.; Martens-Lobenhoffer, J.; Lichtinghagen, R.; Bode-Böger, S.M.; Bavendiek, U.; et al. Markers of endothelial pathology to support detection of atrial fibrillation in embolic stroke of undetermined source. Sci. Rep. 2019, 9, 19424, doi:10.1038/s41598-019-55943-9.

3. Lenaerts, I.; Driesen, R.B.; Hermida, N.; Blanco, N.H.; Holemans, P.; Heidbüchel, H.; Janssens, S.; Balligand, J.-L.; Sipido, K.R.; Willems, R. Role of nitric oxide and oxidative stress in a sheep model of persistent atrial fibrillation. Europace 2013, 15, 754–760, doi:10.1093/europace/eut012.

4. Okawa, K.; Miyoshi, T.; Tsukuda, S.; Hara, S.; Matsuo, N.; Nishibe, N.; Sogo, M.; Okada, T.; Nosaka, K.; Sakane, K.; et al. Differences in endothelial dysfunction induced by paroxysmal and persistent atrial fibrillation: Insights from restoration of sinus rhythm by catheter ablation. Int. J. Cardiol. 2017, 244, 180–185, doi:10.1016/j.ijcard.2017.06.038.

**
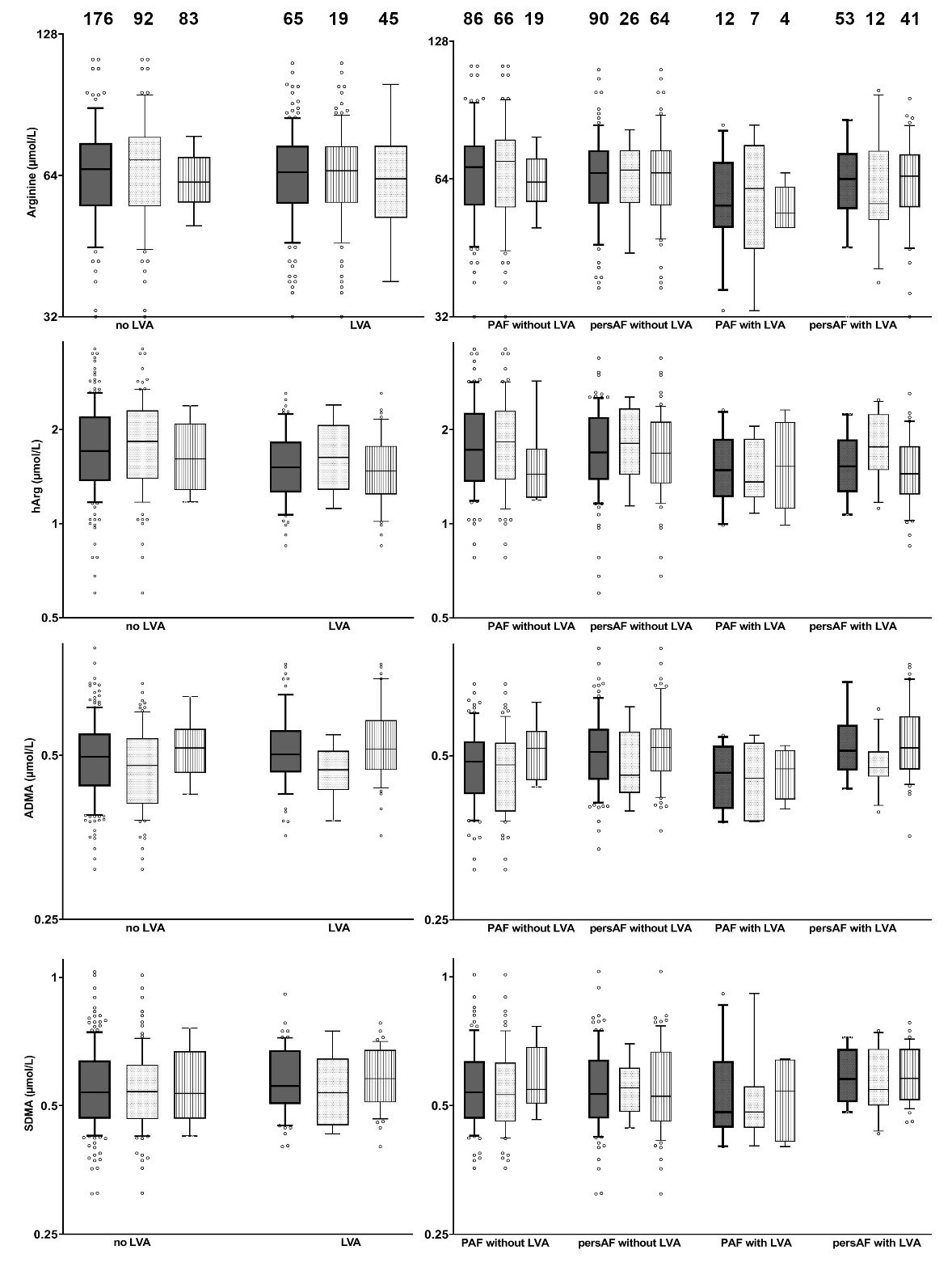
**

**Supplemental Figure 2:** Arginine, homoarginine (hArg), ADMA and SDMA (from top to bottom) (units=µmol/L) in patients with atrial fibrillation. Left side – concentrations in patients without (no LVA) and with low voltage areas (LVA). Right side – comparison of concentrations in patients at different AF progression phenotypes, from left to right: paroxysmal AF (PAF) without LVA, persistent AF (persAF) without LVA, PAF with LVA and persAF with LVA. Dark grey boxes indicate the whole AF cohort, dotted boxes indicate patients in sinus rhythm at the time of blood withdrawal, and lined boxes indicated patients with AF at blood withdrawal. Data are presented box plots with median and whiskers 10-90 percentile. Top of the figure, patient count in every experimental group is shown.
